# Surgical techniques for sciatica due to herniated disc, a systematic review

**DOI:** 10.1007/s00586-012-2422-9

**Published:** 2012-07-20

**Authors:** Wilco C. H. Jacobs, Mark P. Arts, Maurits W. van Tulder, Sidney M. Rubinstein, Marienke van Middelkoop, Raymond W. Ostelo, Arianne P. Verhagen, Bart W. Koes, Wilco C. Peul

**Affiliations:** 1 Department of Neurosurgery, Leiden University Medical Center, Albinusdreef 2, PO Box 9600, 2300 RC Leiden, The Netherlands; 2 Department of Neurosurgery, Medical Center Haaglanden, Lijnbaan 32, 2512 VA The Hague, The Netherlands; 3 Department of Health Sciences, Faculty of Earth and Life Science & EMGO Institute for Health and Care Research, VU University, De Boelelaan 1085, 1081 HV Amsterdam, The Netherlands; 4 Department of Epidemiology and Biostatistics, EMGO-Institute for Health and Care Research, VU University Medical Center, Amsterdam, The Netherlands; 5 Department of General Practice, Erasmus MC, University Medical Center Rotterdam, PO Box 2040, 3000 CA Rotterdam, The Netherlands

**Keywords:** Herniated disc, Sciatica, Surgery, Discectomy, Systematic review

## Abstract

**Introduction:**

Disc herniation with sciatica accounts for five percent of low-back disorders but is one of the most common reasons for spine surgery. The goal of this study was to update the Cochrane review on the effect of surgical techniques for sciatica due to disc herniation, which was last updated in 2007.

**Materials and methods:**

In April 2011, we conducted a comprehensive search in CENTRAL, MEDLINE, EMBASE, CINAHL, PEDRO, ICL, and trial registries. We also checked the reference lists and citation tracking results of each retrieved article. Only randomized controlled trials (RCT) of the surgical management of sciatica due to disc herniation were included. Comparisons including chemonucleolysis and prevention of scar tissue or comparisons against conservative treatment were excluded. Two review authors independently selected studies, assessed risk of bias of the studies and extracted data. Quality of evidence was graded according to the GRADE approach.

**Results:**

Seven studies from the original Cochrane review were included and nine additional studies were found. In total, 16 studies were included, of which four had a low risk of bias. Studies showed that microscopic discectomy results in a significantly, but not clinically relevant longer operation time of 12 min (95 % CI 2–22) and shorter incision of 24 mm (95 % CI 7–40) compared with open discectomy, but did not find any clinically relevant superiority of either technique on clinical results. There were conflicting results regarding the comparison of tubular discectomy versus microscopic discectomy for back pain and surgical duration.

**Conclusions:**

Due to the limited amount and quality of evidence, no firm conclusions on effectiveness of the current surgical techniques being open discectomy, microscopic discectomy, and tubular discectomy compared with each other can be drawn. Those differences in leg or back pain scores, operation time, and incision length that were found are clinically insignificant. Large, high-quality studies are needed, which examine not only effectiveness but cost-effectiveness as well.

## Introduction

Management of sciatica that is caused by a herniated disc varies considerably. Patients are commonly treated in primary care, but a small proportion is referred to secondary care and may eventually undergo surgery if complaints persist for at least 6 weeks. Conservative treatment for sciatica is primarily aimed at pain reduction, either by analgesics or by reducing pressure on the nerve root. However, consensus is lacking as to whether surgery is useful or not in the absence of serious neurologic deficits. There seems to be consensus that surgery is indicated in carefully selected patients with sciatica and presence of a herniated lumbar disc [21, 23, 34]. In most Western countries, especially in the United States, rates of spine surgery are high [14]. The primary rationale of any form of surgery for sciatica due to herniated disc is to relieve nerve root irritation or compression, but the results should be balanced against the likely natural history and the results of conservative care. A recent systematic review indicated that surgery resulted in faster recovery when compared with conservative care, but for the longer term (12 months) no differences were found [31]. The usual indication for surgery is to provide more rapid relief of pain and disability in the minority of patients whose recovery is unacceptably slow [21, 38].

The most common type of surgery is microscopic discectomy, which is defined as surgical removal of part of the disc, performed with the use of an operating microscope or other magnifying tools. Most studies refer to Caspar [11], Yasargil [50], and Williams [49] when discectomy is performed with a microscope; and to Foley and Smith [15] or Greiner-Perth et al. [24] when discectomy is performed with tubular, muscle splitting, retractor systems, and endoscope. However, some have returned to using a microscope, while retaining the less invasive muscle splitting approach of Foley and Smith [15]. There is also uncertainty regarding the relative benefits and harms of different surgical techniques, as was concluded in the 2007 Gibson and Waddell [21] Cochrane review on lumbar disc herniation. This review needs to be updated as several new randomized trials have come to our attention comparing surgical techniques. The objective of this systematic review was to assess the effectiveness of the various surgical techniques for discectomy, such as open, microscopic or tubular discectomy.

## Methods

### Search methods for identification of studies

In the previous Cochrane review for lumbar disc prolapse [20, 21], 40 RCTs, up to January 1st, 2007 were identified. We aimed to update the Cochrane review limited to surgical techniques for lumbar disc herniation with sciatica. For this update, we used the original search strategy in the following databases to identify additional studies:

- Computer-aided searching of MEDLINE, EMBASE, CINAHL, CENTRAL, PEDRO, and ICL from January 2005 to April 2011 using the search strings previously published [19, 21] was performed by the Cochrane Back Review Group. Search strategy is represented in Table 1. No language restrictions were used.

**Table 1:** Search strings and date limits used for different databases for the updated search

Database	Search strings^a^							
MEDLINE	Randomized controlled trial controlled clinical trial randomized placebo drug therapy randomly trial groups [animals not (humans and animals)] dorsalgia	Back Pain backache Low Back Pain (lumbar adj pain) coccyx coccydynia sciatica spondylosis lumbago Spine	Discitis Spinal Diseases (disc adj degeneration) (disc adj prolapse) (disc adj herniation) spinal fusion spinal neoplasms (facet adj joints) intervertebral disk postlaminectomy	Arachnoiditis (failed adj back) Cauda Equina (lumbar adj vertebra^a^) (spinal adj stenosis) [slipped adj (disc^a^ or disk^a^)] [degenerat^a^ adj (disc^a^ or disk^a^)] [stenosis adj (spine or root or spinal)] [displace^a^ adj (disc^a^ or disk^a^)] [prolap^a^ adj (disc^a^ or disk^a^)]	General Surgery Spinal Fusion Laminectomy Intervertebral Disk Displacement Bone Transplantation Intervertebral Disk Chemolysis Chymopapain Diskectomy [(spine^a^ or spinal) adj decompress^a^] laminotomy	Laminoplasty Decompression, Surgical (pedicle adj screw) (facet adj fusion) (lateral adj mass) [(anterior or posterior) adj fusion] (bone adj graft) [fixation adj (spine^a^ or spinal)] [stabili^a^ adj (spine^a^ or spinal)] (pedicle adj fusion)	Foraminotomy (foram^a^ adj stenosis) (lumbar adj body) (vertebra adj body) PLIF GRAF ligamentotaxis (cage adj fusion) (screw adj fusion) (pedicle adj screw)	Chemonucleolysis (cauda adj compress^a^) discectomy diskectomy Laser Therapy Enzymes/tu [Therapeutic Use] (enzyme^a^ adj inject^a^) [(intradisc^a^ or intradisk^a^) adj (steroid^a^ or triamcinolone)] Collagenases/tu [Therapeutic Use]
EMBASE	Clinical Article Clinical Study Clinical Trial Controlled Study Randomized Controlled Trial Clinical Study Double Blind Procedure Multicenter Study	Single Blind Procedure Phase 3 Clinical Trial Phase 4 Clinical Trial crossover procedure placebo allocat$ assign$ blind$	[clinic$ adj (study or trial)] compar$ control$ cross?over factorial$ follow?up placebo$ prospectiv$	Random$ [(singl$ or doubl$ or trebl$ or tripl$) adj (blind$ or mask$)] trial (versus or vs.) human Nonhuman exp ANIMAL Animal Experiment	Dorsalgia back pain LOW BACK PAIN exp BACKACHE (lumbar adj pain) coccyx coccydynia sciatica	ISCHIALGIA spondylosis lumbago SPINE discitis exp Spine Disease (disc adj degeneration) (disc adj prolapse)	(disc adj herniation) spinal fusion spinal neoplasms (facet adj joints) intervertebral disk postlaminectomy arachnoiditis (failed adj back)	Cauda Equina spinal stenosis spine surgery diskectomy discectomy Intervertebral Disk Hernia/su [Surgery]
CINAHL	Back Buttocks	Leg	Back Pain	Back Injuries	Low Back Pain	Sciatica	(low next back next pain)	lbp
CENTRAL	Back Pain dorsalgia backache Low Back Pain lumbar next pain coccyx coccydynia spondylosis Sciatica	Spine Spinal Diseases Lumbago Discitis disc near degeneration disc near prolapse disc near herniation spinal fusion spinal neoplasms	Facet near joints Intervertebral Disk postlaminectomy arachnoiditis failed near back Cauda Equina lumbar near vertebra^a^ spinal near stenosis slipped near (disc^a^ or disk^a^)	Degenerat^a^ near (disc^a^ or disk^a^) stenosis near (spine or root or spinal) displace^a^ near (disc^a^ or disk^a^) prolap^a^ near (disc^a^ or disk^a^) Surgery Spinal Fusion Laminectomy Intervertebral Disk Displacement Bone Transplantation	Intervertebral Disk Chemolysis Chymopapain Diskectomy Decompression, Surgical (spine^a^ or spinal) near decompress^a^ Laminotomy Laminoplasty pedicle near screw facet near fusion	Lateral near mass anterior or posterior) near fusion bone near graft fixation near (spine^a^ or spinal) stabili^a^ near (spine^a^ or spinal) pedicle near fusion foraminotomy foram^a^ near stenosis	Lumbar near body vertebra near body PLIF GRAF Ligamentotaxis cage near fusion screw near fusion pedicle adj screw	Chemonucleolysis cauda adj compress^a^ Laser Therapy (discectomy) or (diskectomy) Enzymes Collagenases enzyme^a^ near inject^a^ (intradisc^a^ or intradisk^a^) near (steroid^a^ or triamcinolone)
PEDro	Clinical Trial :Controlled Clinical Trial Randomized Controlled Trial	Random^a^ placebo^a^ sham	Versus vs “clinical trial”	“controlled trial” double-blind “double blind”	Single-blind “single blind” “BACK”	“BACK PAIN” “LOW BACK PAIN” “LUMBAR SPINE” “LUMBAR VERTEBRAE”	“SCIATICA” “low back pain” “back pain”	Sciatica “LUMBOSACRAL REGION”

^a^For readability we omitted connectors, search fields, explode options and multiple versions of the same search term in one database
- Communication with members of the Cochrane Back Review Group and other international experts.
- Checking reference lists and citation tracking of all papers identified by the above strategies.
- The International Standard Randomized Controlled Trial Number register (ISRCTN) [2], Clinical Trials register [1], USFDA trial register [3] were searched from their beginning at January 1st, 2007 up to April 2011, to identify ongoing studies.

### Criteria for considering studies for this review

Selection criteria for inclusion of studies into the review are given in Table 2. First, we evaluated the studies included in the original Cochrane review against the new criteria (excluding scar tissue and chemonucleolysis trials). At present, chemonucleolysis is neither available nor widely used in most western countries due to safety concerns, namely the risk of allergic reactions to the enzyme that can result in anaphylactic shock—in some patients with fatal consequences. Consequently, we excluded the studies on chemonucleolysis from this update. The comparison of conservative versus surgical treatment was included in a separate, recently published, review [31] and was thus not included here. From the additional electronic search, two review authors (WP, MA) working independently from one another examined titles and abstracts. Full articles were obtained if eligibility could not be ascertained from the title or abstract. Titles and abstracts could be blinded for authors and affiliations, but we did not pursue this with retrieved articles. The two reviewers discussed their selection to meet consensus about inclusions, and a third reviewer was consulted (BK) if consensus was not reached.

**Table 2:** Selection criteria

Types of studies	Randomized controlled trials (RCT)
	No fatal methodological flaw
	Full-text journal article
	Published in a peer reviewed journal
Types of participants	Patients with sciatica due to disc herniation, who have indications for surgical intervention
Types of interventions	Comparisons between all types of surgical intervention were included, such as discectomy, micro-endoscopic-discectomy, automated percutaneous discectomy, nucleoplasty and laser discectomy. Any modifications to these interventional procedures were included, but alternative therapies such as nutritional or hormonal therapies were excluded
Types of outcome measures	All available outcomes were included, but patient centered outcomes were considered of primary interest:
	Pain (Average on VAS or similar, or proportion improved)
	Recovery (Proportion of patients reporting recovery and/or as determined by a clinician)
	Function (Proportion of patients who had an improvement in function measured on a disability or quality of life scale)
	Return to work
	Rate of subsequent low back surgery
	Measures of objective physical impairment: Spinal flexion, improvement in straight leg raise, alteration in muscle power and change in neurological signs
	Adverse complications: Early: Damage to spinal cord, cauda equina, dural lining, a nerve root, or any combination; infection; vascular injury (including subarachnoid hemorrhage); allergic reaction to chymopapain; medical complications; death. Late: Chronic pain, altered spinal biomechanics, instability or both; adhesive arachnoiditis; nerve root dysfunction; myelocele; recurrent symptoms of sciatica due to disc herniation

### Risk of bias assessment

Risk of bias was assessed with the 12-item criteria list recommended by the Cochrane Back Review Group (CBRG) [17]. Criteria are given in Table 3 including operationalization. The items were scored with ‘yes’ (+), ‘no’ (−), or ‘unsure’ (?). Studies were categorized as having a ‘low risk of bias’ when at least six of the 12 criteria were met, and the study had no serious methodological flaws such as extensive loss to follow-up or invalidating trial stop. The risk of bias was assessed independently by two review authors (SR, MvM), who again met to reach consensus. If consensus could not be reached, a third review author (BK) was consulted to resolve the disagreement. The risk of bias assessment in the Cochrane review [20] did not include all items of the current tool used within the Cochrane Back Review Group [17]. Selective outcome reporting, similarity of groups at baseline, and co-interventions were additionally assessed for this review.

**Table 3:** Criteria for risk of bias assessment

	Question	Criteria for “Yes”	Judgment
A	1. Was the method of randomization adequate?	A random (unpredictable) assignment sequence. Examples of adequate methods are coin toss, rolling a dice, drawing of ballots with the study group labels from a dark bag, computer-generated random sequence, pre-ordered sealed envelops, sequentially ordered vials Examples of inadequate methods are alternation, birth date, social insurance/security number, and hospital registration number	Yes/No/Unsure
B	2. Was the treatment allocation concealed?	Assignments are generated by an independent person not responsible for determining the eligibility of the patients. This person has no information about the persons included in the trial and has no influence on the assignment sequence or on the eligibility decision of the patient	Yes/No/Unsure
C	3. Was the patient blinded to the intervention?	The index and control groups are indistinguishable for the patients	Yes/No/Unsure
	4. Was the care provider blinded to the intervention?	The index and control groups are indistinguishable for the care providers	Yes/No/Unsure
	5. Was the outcome assessor blinded to the intervention?	• For patient-reported outcomes with adequately blinded patients • For outcome criteria that supposes a contact between participants and outcome assessors: the blinding procedure is adequate if patients are blinded, and the treatment or adverse effects of the treatment cannot be noticed during examination • For outcome criteria that do not suppose a contact with participants: the blinding procedure is adequate if the treatment or adverse effects of the treatment cannot be noticed during the assessment • For outcome criteria that are clinical or therapeutic events that will be determined by the interaction between patients and care providers, in which the care provider is the outcome assessor: the report needs to be free of selective outcome reporting	Yes/No/Unsure
D	6. Was the drop-out rate described and acceptable?	The number of participants who were included in the study but did not complete the observation period or were not included in the analysis are described and reasons are given and are <20 % for short-term and <30 % for long-term follow-up	Yes/No/Unsure
	7. Were all randomized participants analyzed in the group to which they were allocated?	All randomized patients are reported/analyzed in the group they were allocated to by randomization for the most important moments of effect measurement (minus missing values) irrespective of non-compliance and co-interventions	Yes/No/Unsure
E	8. Are reports of the study free of suggestion of selective outcome reporting?		Yes/No/Unsure
F	9. Were the groups similar at baseline regarding the most important prognostic indicators?	The groups have to be similar at baseline regarding demographic factors, duration and severity of complaints, percentage of patients with neurological symptoms, and value of main outcome measure(s)	Yes/No/Unsure
	10. Were co-interventions avoided or similar?	There were no co-interventions or they were similar between the index and control groups	Yes/No/Unsure
	11. Was the compliance acceptable in all groups?	The compliance with the interventions is acceptable, based on the reported intensity, duration, number and frequency of sessions for both the index intervention and control intervention(s). For single-session interventions (for ex: surgery), this item is irrelevant	Yes/No/Unsure
	12. Was the timing of the outcome assessment similar in all groups?	Timing of outcome assessment was identical for all intervention groups and for all important outcome assessments	Yes/No/Unsure

### Data collection and analysis

Included studies were categorized under separate comparisons with clinically homogeneous characteristics. An a priori list of items was used for the data extraction, consisting of both descriptive data (e.g., study population, type of interventions, outcome parameters used) and quantitative data regarding the primary and secondary outcome measures. One reviewer (WJ) extracted the data and entered the data into Review Manager (RevMan, Version 5.1. The Cochrane Collaboration, 2011). We aimed at analyzing the parameters of surgical morbidity (operation duration (min), blood loss (ml), incision length (mm), length of stay (days)), and clinical outcomes (low back pain (VAS), leg pain (VAS), and other clinical outcomes (for example, Oswestry, JOA, SF-36, Return to Work). Pain (low back or leg) is regarded the primary outcome. The main endpoint for clinical outcome was defined as 2 years, Where possible, an attempt was made to categorize patients according to their symptom duration (less than 6 weeks,6 weeks to 6 months, more than 6 months), by their response to previous conservative therapy and type of disc herniation. The overall quality of the evidence was graded as ‘High’, ‘Moderate’, ‘Low’, or ‘Very low’, according to the GRADE approach [25]. This means that the overall quality of evidence was initially regarded as ‘High’, but was downgraded if there were limitations in design according to the risk of bias assessment, inconsistency, indirectness, imprecision, or publication bias. For comparisons with only one reported outcome, or with only one study, no grading was performed. With sufficient clinically and statistically homogeneous and sufficiently comparable and adequately reported outcomes, data were pooled and forest plots were generated using Revman. Random effects estimates were used for all analyses. To identify publication bias, funnel plots were examined. Because of the limited clinical value, no pooled analyses were performed for low and very low quality of evidence, conflicting evidence, or indirect evidence.

## Results

### Search and selection results

Seven of the 42 studies from the original Cochrane review were included. We excluded 30 studies because the interventions evaluated did not meet our new, limited, selection criteria pertaining to surgical techniques. Studies were excluded because they examined some form of chemonucleolysis (18), because they compared conservative with surgical interventions (4), or because they examined any type of barrier membrane for prevention of scar tissue (8). Additionally, we excluded five studies from the original review, being two conference proceedings [37, 42]; one summary of two included studies [18]; and two studies with a fatal flaw due to trial stop after interim analysis [12], and due to trial stop after only 10 % of the sample size [26, 27].

**Fig. 1:**
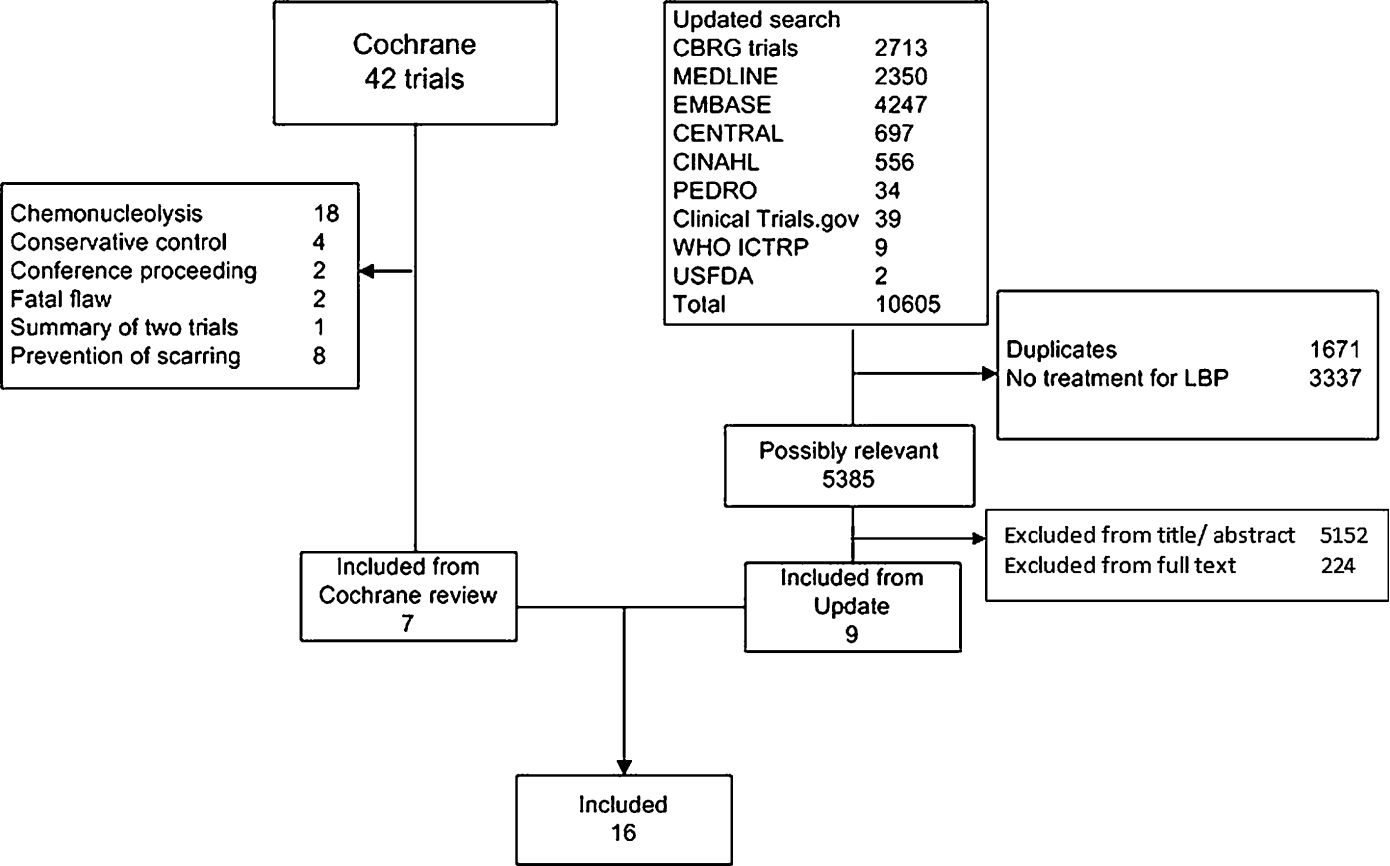
Flow chart for inclusion of studies

We identified seven additional studies published since publication of the previous review [6, 10, 16, 39–41, 43] and two studies that were published in 2006 or before but for unclear reasons not included in the Cochrane review [32, 46]. The study from Arts et al. [6] was reported in four additional publications with analysis of effect modifiers [5], assessment of muscle injury [4], 2-year results [7], and cost-effectiveness analysis [47]. Two additional papers reported long-term follow-up of the Thome et al. [44] study concerning clinical [9] and radiological [8] results. Details of the search are presented in Fig. 1. There were no ongoing studies found. A total of 16 studies were included.

### Risk of bias assessment

Risk of bias assessments are reported in Table 4. Four studies were considered to have a low risk of bias study (six positive items). Randomization was adequate in half of the studies and allocation concealment was adequately described in only three studies. Outcome assessor blinding and patient blinding was used in a quarter of the studies. Selective reporting is doubtful in most studies, as rarely a prepublished protocol could be found. Compliance is by definition graded ‘Yes’ (+), as this review deals with a surgical technique. Quality of evidence for the separate outcomes for all comparisons is given in Table 5.

**Table 4:** Risk of bias assessment of included studies

Comparison	A1	B2	C3	C4	C5	D6	D7	E8	F9	F10	F11	F12
Study, Year	Randomisation	Allocation concealment	Patient blinding	Surgeon blinding	Outcome blinding	Drop-outs	ITT	Selective reporting	Baseline	Co interventions	Compliance	Outcome timing
Open versus minimal invasive discectomy												
Henriksen 1996 [28]	+	−	+	−	+	+	?	?	+	+	+	+
Hermantin 1999 [29]	+	?	−	−	−	+	+	?	+	?	+	+
Huang 2005 [30]	?	?	−	−	−	?	?	?	?	+	+	−
Katayama 2006 [32]	+	?	?	−	?	?	?	?	?	?	+	−
Lagarrigue 1994 [33]	+	+	−	−	+	?	−	?	+	?	+	?
Tullberg 1993 [45]	?	?	−	−	−	+	−	?	+	?	+	+
Tureyen 2003 [46]	+	?	?	?	?	−	?	?	+	?	+	+
Teli 2010 [43]	?	?	−	−	−	+	−	?	+	+	+	+
Different techniques of minimal invasive discectomy												
Arts 2009 [6]	+	+	+	−	+	+	+	?	+	?	+	+
Brock 2008 [10]	−	−	+	−	+	?	?	?	−	?	+	?
Franke 2009 [16]	?	?	?	−	?	?	?	?	+	?	+	+
Mayer 1993 [35]	?	?	−	−	−	+	?	?	+	?	+	+
Righesso 2007 [39]	?	?	−	−	−	?	?	?	+	?	+	+
Ryang 2008 [40]	?	?	−	−	−	?	?	?	+	?	+	−
Shin 2008 [41]	+	−	−	−	−	?	?	?	?	?	+	+
Thome 2005 [44]	+	+	?	−	?	+	−	?	+	?	+	+

**Table 5:** Quality of evidence for reported outcomes

Comparison	Studies	Patients	Grade limitations					Summary of findings		Quantitative
Outcome			Publication bias	Inconsistency	Indirectness	Imprecision	Risk of bias	Effect	Quality	Pooled effect
Open (OD) versus minimal invasive discectomy (MID) 6 studies										
Surgery duration (min)	6	612	+	+	+	+	−	OD < MID	Moderate	MD 12.2 (2.20 to 22.3)
Length of stay (days)	5	452	+	+	+	+	−	OD <> MID	Moderate	MD −0.06 (−0.10 to +0.21)
Blood loss	2	179	−	+	+	−	−	OD ? MID	Very low	
Incision	3	353	+	−	+	+	−	OD > MID	Low	
Leg pain (mm VAS)	4	453	+	+	+	+	−	OD > MID	Moderate	MD −2.01 (−3.44 to −0.57)
Back pain (mm VAS)	3	419	−	−	+	+	−	OD ? MID	Very low	
Return to work	3	254	?	−	+	+	−	OD ? MID	Very low	
Tubular (TD) versus microscopic discectomy (MID) 7 studies										
Surgery duration (min)	6	718	+	−	+	−	−	TD ? MID	Very low	
Blood loss	3	130	−	+	+	−	−	TD ? MID	Very low	
Length of stay (days)	4	528	+	+	+	−	−	TD <> MID	Low	
Incision	3	260	+	+	+	?	−	TD < MID	Low/Moderate	SD sparsely reported
Leg pain (mm VAS)	3	548	−	−	+	+	−	TD ? MID	Very low	
Back pain (mm VAS)	4	703	+	−	+	+	−	TD <> MID	Low	
Oswestry	3	225	?	+	+	−	−	TD ? MID	Very low	
SF36	3	548	?	+	+	+	−	TD ? MID	Low	

^a^< or > Effect is superior for one of both treatments; <> None of either treatments is superior; ? unclear relative effectiveness due to conflicting results

*MD* Mean difference, *OR* odds ratio

### Effects of interventions

We distinguished comparisons between open and minimal invasive discectomy and comparisons between different techniques for minimal invasive discectomy. It was not possible to analyze patients according to duration of their symptoms, previous conservative treatment, type of disc herniation, or indications for surgery, as too few data were available. Many studies provided limited information on complications. All quality of evidence was downgraded because of the risk of bias in the studies: further downgrading is noted in the text.

### Open versus minimal invasive discectomy

Eight studies compared open discectomy (OD) versus minimal invasive techniques such as use of loupe magnification or microscope (MID), video-assisted microscopic discectomy (VAMD) or micro-endoscopic discectomy (MED). One of the studies compared three types of surgery: open, microscopic, and micro-endoscopic discectomy and could be included in three comparisons. Characteristics of included studies are presented in Table 6. The results of these studies are given in Table 7.

**Table 6:** Characteristics of the included studies comparing open versus microscopic and comparable discectomy

Author, year	Sample size	Female (%)	Average age (range/SD)	Participants	Interventions	Outcomes	Follow-up
Henriksen 1996 [28]	79	37	41 (30–48)	HNP, 20 to 60 years, failed conservative therapy (bed rest, analgesics,muscle relxers, physiotherapy), myelogram, CT verified	Open (standard) discectomy (OD) Microscopic discectomy (MD)	Incision, OP time, LOS Pain medication Pain (VAS)	2, 4, 6 days 2, 4, 6 weeks
Hermantin 1999 [29]	60	35	40 (15–67)	Low back pain and radicular symptoms, confirmed by imaging, due to single level intercanalicular HNP at L2-S1. < 50 % canal, no osseous or ligamenteous stenosis, failed conservative treatment, back pain > leg pain, no severe disc height loss	Open laminectomy and discectomy (OD) Video assisted arthroscopic microdiscectomy (VAMD)	Self evaluation Physical examination Return to function Pain (Houde) Satisfaction Return to work	2 weeks 3, 6 months 1, 2 years
Huang 2005 [30]	22	32	39.4 (10.9)	Failed conservative treatment (3 months), OR Acute attack of intractable back and leg pain, no improvement 1–2 weeks bedrest. No motor deficit or sphincter disturbance	Open discectomy (OD) Microendoscopic discectomy (MED)	OP time, Blood loss, LOS Interleukines and CRP Pain (VAS) MacNab	18.9 months (10–25)
Katayama 2006 [32]	119	36	37 (14–65)	Primary surgery for HNP	Open (macro) discectomy (OD) Microscopic discectomy (MD)	OP time, Blood loss, LOS Pain medication JOA VAS back pain VAS sciatica Complications/reoperations	2.67 years (1–4)
Lagarrigue 1994 [33]	80	49	43 (15–80)	HNP with sciatica, failed conservative treatment (3 months), CT confirmed. No paralysis, stenosis, degenerative changes	Open discectomy (OD) Microscopic disectomy (MD)	MacNab OP time, LOS RTW	14.9 months (12–18)
Teli 2010 [43]	240	34	39.3 (27–61)	Symptomatic, single-level HNP, 18–65 years, concordant neurological signs, failed conservative treatment (6 weeks, pain medication, epidural steroids), no additional spinal disorders	Open discectomy (OD) Microscopic discectomy (MD) Microendoscopic discectomy (MED)	OP time, Incision Back pain (VAS) Leg pain (VAS) Oswestry disability SF36 Cost	10 days 6, 12, 24 months
Tullberg 1993 [45]	60	35	39 (17–64)	Single lumbar disc herniation, failed conservative treatment (2 months), CT verified	Open (standard) discectomy (OD) Microscopic discectomy (MD)	OP time, Blood loss, LOS Back pain (VAS) Leg pain (VAS) Satisfaction	3 weeks 2, 6, 12 months
Tureyen 2003 [46]	114	43	41.6 (18–61)	Lumbar disc herniation, leg pain, MRI verified	Laminectomy and macrodiscectomy (OD) Microscopic discectomy (MD)	OP time, LOS, incision Radicular pain (VAS) Muscle strength (MRC) Sensation Reflex	10 days 1 month 1 year

*LOS* Length of stay, *RTW* Return to work, *JOA* Japanese Orthopaedic Association score

**Table 7:** Results of the included studies: open discectomy versus microscopic discectomy for disc herniations with sciatica

Author, year	Group	Crossover (*n*, %) to other group	Surgical morbidity				Pain (VAS in mm) (sd, range) at 2 years	Recovery/Clinical outcome at 2 years^#^	Qualitative conclusions
			OP time	Blood loss (gr or ml)	LOS (days)	Incision			
Henriksen 1996 [28]	Open (standard) discectomy (OD)	0	35 (30–40)	–	4.6 (3–7)	Skin: 71 (2.5) Fascia: 70 (2.0)	Not extractable, no difference	–	Shorter incision does not affect LOS or pain
	Microscopic discectomy (MD)	0	48 (37–60)		5.2 (3–6)	Skin: 72 (2.5) Fascia: 31 (2.5)			
Katayama 2006 [32]	Open (macro) discectomy (OD)	?	40 (12)	39 (11)	8.3 (0.8)	–	VAS lumbar at 2.7 years: 16 (7) VAS sciatica at 2.7 years: 13 (5)	JOA at 2.7 years: 27 (1)	Small differences in OP time, blood loss, hospitalization. No difference in analgesics. Long term: Small difference in VAS lumbar pain. No differences in VAS sciatica or JOA
	Microscopic discectomy (MD)	?	45 (8)	25 (9)	8.5 (2.3)		VAS lumbar at 2.7 years: 12 (04) VAS sciatica at 2.7 years: 12 (04)	JOA at 2.7 years: 27 (1)	
Lagarrigue 1994 [33]	Open discectomy (OD)	?	60	–	6.5	–	–	MacNab at 14.9 months 90 % RTW at 14.9 months: 77 days	No difference in clinical outcome, operating time, hospital stay, or return to work
	Microscopic discectomy (MD)	?	65		6.2			MacNab at 14.9 months: 90 % RTW at 14.9 months: 94 days	
Tullberg 1993 [45]	Open (standard) discectomy (OD)	0	46 (20–95)	45 (10–200)	2.3 (1–5)	–	VAS leg pain at 1 year: 23 VAS back pain at 1 year: 18	Sick leave: 10.1 weeks Recovery: 90 %	No differences in bleeding, complications, LOS, sick leave and clinical outcomes (pain and recovery)
	Microscopic discectomy (MD)	0	60 (25–90)	47 (10–200)	2.5 (1–3)		VAS leg: 21 VAS back: 16	Sick leave: 10.4 weeks Recovery: 86 %	
Tureyen 2003 [46]	Laminectomy and macrodiscectomy (OD)	?	25 (20–90, 7.07)	–	1 (1–2)	6 (5–7)	Radicular pain (VAS): 14 (0–30)	RTW at 4 weeks: 54 %	Differences in incison and operative time and earlier return to work, and analgesics use. No further differences
	Microscopic discectomy (MD)	?	54 (25–95, 5.25)		1 (1–2)	4 (3–5)	Radicular pain (VAS): 12 (0–30)	RTW at 4 weeks: 88 %	
Teli 2010 [43]	Open discectomy (OD)	?	36 (10)	–	–	Skin: 23	VAS leg pain: 20 (10) VAS back pain: 10 (10)	Oswestry: 14 (5)	Comparable outcome, MED more costly and more complications
	Microscopic discectomy (MD)	?	43 (8)			Skin: 22	VAS leg pain: 20 (10) VAS back pain: 20 (10)	Oswestry: 13 (5)	
	Microendoscopic discectomy (MED)	?	56 (12)			Skin: 10	VAS leg pain: 20 (10) VAS back pain: 20 (10)	Oswestry: 15 (5)	
Huang 2005 [30]	Open discectomy (OD)	0	72.1 (17.8)	190 (115)	5.92 (2.39)	6.3 (0.98)	VAS at 18.9 Months 14 (01,10–30)	MacNab at 18.9 months: 90 %	Surgical trauma is less with MED than OD. Clinical outcomes are comparable
	Microendoscopic discectomy (MED)	1^a^	109 (35.9)	87.5 (69.4)	3.57 (0.98)	1.86 (0.13)	VAS 15 (02, 10–20)	MacNab at 18.9 months: 91.6 %	
Hermantin 1999 [29]	Open discectomy (OD)	0	–	–	–	–	According to Houde: 1.9	RTW/resume normal activity: 49 days Good outcome: 93 %	Same satisfactory outcome; VAMD shorter disability
	Video assisted MD (VAMD)	0					According to Houde: 1.2	RTW/resume normal activity: 27 days Good outcome: 97 %	

*LOS* Length of stay, *RTW* Return to work, *JOA* Japanese Orthopaedic Association score

^a^One patient insisted on OD; the text is ot clear if this patient was randomized to MED or not randomized at all

**Fig. 2:**
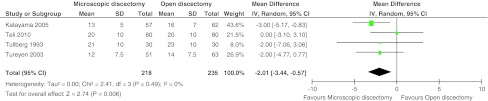
Forest plot for VAS leg pain between microscopic discectomy and open discectomy

**Fig. 3:**
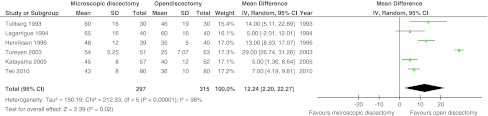
Forest plot for operating time between microscopic discectomy and open discectomy

**Fig. 4:**
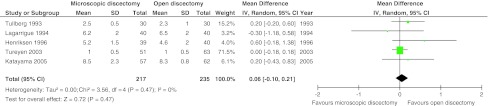
Forest plot for length of stay between microscopic discectomy and open discectomy

Six studies with 612 patients (five with high risk of bias) compared the classical open (or standard- or macro-) discectomy with microscopic discectomy [28, 32, 33, 43, 45, 46]. Leg pain was reported in four studies with 453 patients. There was moderate quality of evidence that postoperative leg pain was statistically significantly less for microscopic discectomy by 2.01 mm (95 % CI 0.57–3.44; *p* = 0.006; see Fig. 2). The follow-up of these studies ranged from 1 to 2.7 years. A higher proportion of patients with return to work was found at 4 weeks for microscopic discectomy [46] in one study (*n* = 114), whereas two other studies (*n* = 140) found no difference at 10 weeks [45] and 15 months [33]. All six studies found an increased operating time for microscopic discectomy with a pooled effect of 12.2 min (95 % CI 2.20–22.3; *p* = 0.02; moderate quality of evidence; see Fig. 3). Length of stay was reported in five studies with 452 patients, but no differences were found. The mean difference was 0.06 days in favor of open discectomy (95 % CI −0.10 to +0.21 days; *p* = 0.47; moderate quality of evidence; see Fig. 4). Blood loss was reported in two studies, in one study (*n* = 119) microscopic discectomy resulted in less blood loss [32], while in the other study (*n* = 60) there was no difference [45]. Length of incision was reported in three studies (*n* = 353) and found to be shorter for microscopic discectomy in two studies [28, 46]. The quality of evidence for blood loss had to be downgraded due to risk of bias, publication bias and imprecision and was ‘very low’. Quality of evidence for incision was ‘low’ due to risk of bias and inconsistency (Table 5). Therefore, these results were not pooled.

Two studies compared open with micro-endoscopic discectomy (MED) [30, 43]. Huang et al. [30] reported results of a very small, high risk of bias, study (*n* = 22). There were no differences in leg pain severity and MacNab criteria between the groups. The MED group had shorter postoperative hospital stay (3.6 vs. 5.9 days) and less intraoperative blood loss (88 versus 190 ml) compared with the open discectomy group, but duration of the operation was longer (109 vs. 72 min). Teli et al. [43] showed in a larger high risk of bias study (*n* = 220) that the MED group compared with open and microscopic discectomy suffered more dural tears (6/70, 2/72, 2/70, respectively), root injuries (2/70, 0/72, 0/70, respectively), and recurrent herniations (8/70, 3/72, 2/70, respectively).

One low risk of bias study (*n* = 60) found that patients who had received video-assisted arthroscopic microdiscectomy had similar satisfactory outcomes (based on self evaluation, return to work, and physical exam) compared with open laminotomy and discectomy, but patients who had had an arthroscopic microdiscectomy had a shorter duration of postoperative disability (27 vs. 49 days) and had a lower narcotic use score [29].

### Various types of microdiscectomy

Nine studies with 1,047 patients evaluated different approaches for less invasive discectomy, such as use of loupe magnification or microscopic discectomy (MD), micro-endoscopic discectomy (MED), tubular microscopic discectomy, microscopic assisted percutaneous nucleotomy (MPN), minimal access trocar/microsurgical microdiscectomy (MAMD), percutaneous endoscopic discectomy or sequestrectomy. We analyzed the comparisons between these techniques, keeping the differences in muscle damage and differences in use of microscope or endoscope in mind. Characteristics of included studies are presented in Table 8. The results of these studies are given in Table 9.

**Table 8:** Characteristics of the included studies comparing different techniques of minimal invasive discectomy

Author, year	Sample size	Female (%)	Average age (range/SD)	Participants	Interventions	Outcomes	Follow-up
Arts 2009 [4, 6, 7]	328	47	41.5 (18–70, 10.8)	HNP + persistent radicular pain (>8 weeks). Unsuccessful conservative treatment. The Netherlands	Transmuscular tubular microscopic discectomy Conventional microscopic discectomy	OP time, blood loss, LOS Roland-Morris (RMDQ) Back pain (VAS) Leg pain (VAS) SF36 Sciatica frequency and bothersome (SFBI) Recovery (self-reported) Muscle injury Cost effectiveness	2, 4, 6, 8, 12, 26, 38 weeks 1, 2 years
Brock 2008 [10]	125	49	51 (20–79)	First time lumbar microdiscectomy, failed conservative treatment (12 weeks). Germany	Subperiosteal microscopic discectomy Transmuscular microscopic discectomy	Leg Pain (VAS) Back pain (VAS) Oswestry LOS Analgesics use	1, 6 days before discharge
Franke 2009 [16]	100	40	44 (21–72, 11.7)	Disc dislocations grades 3–5 (Kramer), no lateral HNP, no protrusions	Microscopic discectomy Microscopically Assisted Percutaneous Nucleotomy	OP time, LOS RTW Oswestry Back pain (VAS) Leg pain (VAS) Neurological deficits	8 weeks 6, 12 months
Mayer 1993 [35]	40	35	41.3 (12–63, 10.2)	Previous unsuccessful conservative treatment (time period not stated). Only small “non-contained” disc herniations included. Berlin, Germany	Percutaneous Endoscopic Discectomy Micro-surgical discectomy	OP time Patient rating Surgeon rating	2 years
Righesso 2007 [39]	40	43	43.9 (11.5)	Posterolateral HNP and persistence of sciatica, failed conservative treatment (4–8 weeks) with rest, analgesia, NSAIDs and physical therapy. MRI verified. Brazil	Microendoscopic discectomy Open discectomy with loupe	OP time, Blood loss, LOS Incision Pain (VAS) Oswestry Neurological status	12 h 1, 3, 6, 12, 24 months
Ryang 2008 [40]	60	47	38.7 (21–69, 10.3)	Single level virgin HNP; typical monoradicular symptoms, sciatica > > lower back pain, failed conservative treatment (8 to 12 weeks). Germany	Minimal access microscopic discectomy Open microscopic discectomy	Pain: VAS (10 cm) Oswestry SF-36	16 months (6–26)
Shin 2008 [41]	30	60	45.4 (14.6)	Single-level unilateral HNP, failed conservative treatment (> 6 weeks), CT or MRI verified. Korea	Microendoscopic discectomy Microscopic discectomy	Back pain (VAS) Leg pain (VAS) Blood enzymes (CPK, LDH)	1, 3 and 5 days
Teli 2010 [43]				See Table 6			
Thome 2005 [44]	84	44	(18–60)	Single level HNP, failed conservative treatment, 18-60 years, MRI verified. Mannheim, Germany	Disc sequestrectomy Microscopic discectomy	OP time, blood loss Patient satisfaction index Prolo scale SF-36 Low back pain (VAS) Sciatica (VAS) Repeat surgery	Discharge 4–6 months 12–18 months 2-years

*LOS* length of stay, *RTW* Return to work

**Table 9:** Results of the included studies: Different techniques for minimal invasive discectomy for disc herniations with sciatica

Author, year	Group	Crossover (*n*, %) to other group	OP time (mins)	Blood loss (gr or ml)	LOS (days)	Incision (cm)	Pain (VAS in mm) (sd, range) at 2 years	Recovery/Clinical outcome at 2 years^#^	Qualitative conclusions
Arts 2009 [6]	Conventional microdiscectomy (MD)	2	36 (16)	% <50 ml: 85	3.3 (1.1)	–	VAS leg pain 14.0 (se 1.8) VAS back pain 19.4 (se 1.9)	RMDQ: 3.7 (se 0.5) SF 36: physical 82.4 (se 1.8)	TD bit more leg and back pain, other outcomes not different. Not less muscle injury
	Transmuscular tubular discectomy (TD)	2	47 (22)	% < 50 ml: 92	3.3 (1.2)		VAS leg pain 15.3 (se 1.7) VAS back pain 23.5 (se 1.9)	RMDQ: 4.5 (se 0.5) SF36: 78.9 (se 1.7)	
Brock 2008 [10]	Subperiosteal microdiscectomy (MD)	?	–	–	–	–	VAS leg pain discharge: 14 VAS back pain discharge: 17	Oswestry at discharge: 20	Analgesics consumption less with transmuscular approach. Pain and Oswestry similar
	Transmuscular discectomy (TD)	?					VAS leg pain discharge: 9 VAS back pain discharge: 12	Oswestry at discharge: 25.7	
Franke 2009 [16]	Microscopic Discectomy (MD)	0	56.3 (19.2)	–	4.9	–	–	–	Shorter OP time and quicker recovery at experienced center for MAPN. No clinical or complication rate differences
	Percutaneous Nucleotomy (MAPN)	0	41.8 (15.5)		3.8				
Mayer 1993 [35]	Micro-discectomy (MD)	0	58.2 (15.2)	–	–	–	–	Clinical score: 7.67 (1.9)	Clinical results comparable, in some respects percutaneous superior
	Percutaneous Endoscopic Discectomy (PED)	0	40.7 (11.3)					Clinical score: 8.23 (1.3)	
Righesso 2007 [39]	Open discectomy with loupe (MD)	?	63.7 (15.5)	40 (11–450)	26 (16–72) h	2.6 (0.4)	VAS: 0 (0–60)	Oswestry: 10 (0–30)	MD longer LOS and incision, MED longer OP time. No clinical differences
	Microendoscopic discectomy (MED)	?	82.6 (21.9)	50 (10–700)	24 (11–72) h	2.1 (0.2)	VAS: 10 (0–30)	Oswestry: 10 (0–22)	
Ryang 2008 [40]	Open microscopic discectomy (MD)	0	92 (33–150, 28.6)	63.8 (0–300, 86.8)	4.4 (1–15, 2.8)	4–5	VAS Back pain at 16 months: 21 (0–98, 24)	Oswestry at 16 months: 12 (0–86, 18.8) SF 36: at 16 months physical 47.5 (9.4) and mental 51.9 (7.8)	No differences
	Minimal access microdiscectomy (MAD)	0	82 (37–120, 25.1)	26.2 (0–100, 29.7)	4 (2–14, 2.3)	1.6	VAS Back pain at 16 months: 21 (0–75, 24)	Oswestry at 16 months: 12 (0–46, 14) SF36: Physical 47.6 (10.7) and mental 44 (13.2)	
Shin 2008 [41]	Microscopic discectomy (MD)	?	47 (5)	34 (11)	–	–	VAS Back at 5 days: 36 (11) VAS leg at 5 days: 24 (21)	–	MED faster relief of back pain within the first 5 days post-operatively, but no differences in leg pain
	Microendoscopic discectomy (MED)	?	49 (5)	35 (9)			VAS Back at 5 days: 19 (11) VAS leg at 5 days: 25 (16)		
Teli 2010 [43]	Microscopic discectomy (MD)	?	43 (8)	–	–	2.2	VAS leg pain: 20 (10) VAS back pain: 20 (10)	SF36: 13 (5)	MED more costly and more complications
	Microendoscopic discectomy (MED)	?	56 (12)			1.0	VAS leg pain: 20 (10) VAS back pain: 20 (10)	SF 36: 15 (5)	
Thome 2005 [44]	Microdiscectomy (MD)		38.2 (10.3)	78.2 (61.6)	–	–	–	–	Clinical results favoring sequestrectomy
	Disc sequestrectomy (DS)		32.6 (13.8)	67.0 (85.4)					

Average given, with in brackets range (xx–xx) or sd (xx) or both (xx–xx, xx), or se when indicated. Follow-up as in column headers, unless indicated otherwise

*LOS* Length of stay

Seven (six with high risk of bias) studies with 923 patients compared tubular discectomy with conventional microscopic discectomy [6, 10, 16, 39–41, 43]. Of these, four used an endoscope [39–41, 43]. One study found a faster improvement in pain scores for tubular discectomy before discharge [41], while the only low risk of bias study found a slightly better pain score for conventional discectomy at 2 years [6]. All other outcomes for pain as measured with VAS, for functioning as measured with Oswestry or Roland-Morris score, or for quality of life measured with SF36 were not significantly different between the two surgical techniques. In Shin et al. [41], baseline values for back pain were not comparable. In one study, the post-operative analgesic consumption was significantly less in the tubular discectomy group [10]. Inconsistent results were found for operative morbidity. There was low to moderate quality of evidence for incision length (Table 5) and this was consistently shorter for tubular discectomy in all three studies (*n* = 260) that reported this outcome [39, 40, 43] (Table 9). However, results could not be pooled due to sparse data on variation (SD). The quality of evidence for surgery duration, blood loss and length of stay was ‘Low’ to ‘Very low’ due to risk of bias in the studies, imprecision, inconsistency, and/or publication bias, so no further meta-analyses could be performed (Table 5). Two studies (*n* = 368) of the six studies (*n* = 718) reporting operative time found a longer duration for tubular discectomy [6, 39], while one study (*n* = 100) found a shorter duration [16]. No differences were found for blood loss in three studies. Length of stay was longer (2 h to 1.1 days) for conventional microscopic discectomy in two of four studies [16, 39].

One high risk of bias study [35] with 40 patients compared percutaneous endoscopic discectomy (cannula inserted into the central disc) with microscopic discectomy. This study showed comparable clinical outcomes after the two procedures but contained a small sample size.

One low risk of bias study [44] with 84 patients compared clinical outcomes and recurrence rates after sequestrectomy (removal of only the sequestration while leaving the remaining disc intact) and standard microdiscectomy (removing the herniated material and resection of disc tissue from the intervertebral space). There were no statistically significant differences in back and leg pain and quality of life up to 2 years of follow-up [9].

## Discussion

Limited quality and amount of evidence were found that microscopic discectomy results in at least an equal clinical outcome compared with open discectomy. There was only moderate quality evidence that microscopic discectomy resulted in a clinically irrelevant reduction of leg pain of 2 mm (on a 100-mm scale) compared with open discectomy at 1–2 years, which is regarded clinically relevant at minimal 15 mm according to Ostelo et al. [31]. For back pain and return to work, the evidence is of very low quality and suffers from inconsistency, risk of bias, and possibly publication bias. Concerning operative morbidity, microscopic discectomy results in decreased incision length compared with open discectomy while the surgical duration increased with microscopic discectomy.

When tubular discectomy was compared with microscopic discectomy, there were conflicting results for the main outcomes of surgical duration and for back pain from discharge to 24 months. Leg pain, Oswestry score, and SF36 scores could not be reliably estimated because of the few studies reporting these outcomes. In principle, the microscope provides better illumination and facilitates teaching. The choice of open or (type of) microscopic discectomy at present probably depends more on the training and expertise of the surgeon, and the resources available, than on scientific evidence of efficacy. However, it is worth noting that some form of magnification is now used almost universally in major spinal surgical units to facilitate vision. New techniques should only be used under controlled circumstances in a clinical trial that compares against microscopic discectomy, open discectomy or conservative interventions. Use of the more costly microsurgical techniques with comparable clinical outcomes would be justified if the advantages of reduced surgical morbidity were proven with at least an equal clinical outcome. A non-inferiority design would have been applicable to answer this question, but so far, has not been used, and test of non-inferiority was not anticipated in this review. A secondary cost–utility analysis on one trial comparing conventional versus MED [6] showed non-significant higher cost for the MED technique [47].

The place for other forms of discectomy is unresolved. Studies of automated percutaneous discectomy and laser discectomy suggest that clinical outcomes following treatment are at best fair and probably worse than after microscopic discectomy, although the importance of patient selection is acknowledged. There are no studies examining intradiscal electrotherapy, coblation or fusion as a treatment for sciatica due to disc herniation.

Many of the studies had major design weaknesses. For example, some of the studies had a very small sample size, which was only complicated by the fact that many of these had not performed a sample size calculation; therefore, the possibility for type II error cannot be ruled out. Methods and published details of randomization were often poor and there was lack of concealment of treatment allocation. Given the nature of surgical interventions, surgeon blinding was not possible. Blinded assessment of outcome was generally feasible yet often not even attempted. There were few clinical outcomes meeting standardized requirements [13]. It is remarkable that leg pain was only reported in about half of the studies, while this could be regarded as the main reason for performing surgery in these patients. Some of the assessments were made by the operating surgeon or by a resident or fellow beholden to the primary investigator, thus introducing assessor bias. Although most of the studies had follow-up rates of at least 90 %, there was often unclear early code break or crossover of patients not properly described, let alone allowed for in the analysis or presentation of results. These defects of study design introduced considerable potential for bias. Most of the conclusions of this review are based upon 6- to 12-month outcomes and there is a general lack of information on longer-term outcomes. Only a minority of the studies, especially the older ones, presented 2-year follow-up results as recommended for surgical studies.

To put our results into perspective, our systematic review was compared with the three reviews that studied different surgical techniques and which were published in 2009 [22, 36, 48]. These reviews have serious limitations in methodology. McGirt et al. [36] and Watters and McGirt [48] use the same search strategy and methodology and can be regarded as the same review with a different outcome parameter (overall outcome and recurrent disc herniation). Both randomized and non-randomized controlled trials as well as case series are included, thus making it difficult to decipher the effect of surgery. In both reviews there are conflict of interest issues [36, 48]. Both reviews do not use an accepted pooling method and should not be used for decision analysis. For example, McGirt et al. [36] include the comparative studies and the case series and analyze both study designs in the same analysis. Gotfryd and Avanzi [22] include ten (quasi-)randomized studies comparing classical discectomy, microdiscectomy, and/or endoscopic discectomy. They only evaluated randomization and allocation concealment as possible risk of bias items. This limits the possibility to assess the effect of other possible sources of bias in the comparisons, such as lack of blinding and poor attrition. They concluded that microsurgical and endoscopic techniques are only superior with regard to blood loss, hospital stay end systemic repercussions, but not for satisfaction, pain or other clinical parameters. To conclude, we believe our review produces reliable and valid results because no conflict of interest is present and the use of the Cochrane methods guarantees high quality.

## Conclusion

Implications for practice: due to the limited amount and quality of evidence, no firm conclusions on effectiveness of the current surgical techniques, being open discectomy, microscopic discectomy, and tubular discectomy, compared with each other can be drawn. Those differences in leg or back pain scores, operation time, and incision length that were found are clinically insignificant. Therefore, the surgical strategy in the treatment of lumbar disc herniation should be based on preferences of patients and surgeons rather then outcome measures.

Implications for research: large, high-quality studies are needed, which examine not only effectiveness but cost-effectiveness as well.
